# Magnetic Drug Targeting: A Novel Treatment for Intramedullary Spinal Cord Tumors

**DOI:** 10.1038/s41598-018-29736-5

**Published:** 2018-07-30

**Authors:** Pouyan Kheirkhah, Steven Denyer, Abhiraj D. Bhimani, Gregory D. Arnone, Darian R. Esfahani, Tania Aguilar, Jack Zakrzewski, Indu Venugopal, Nazia Habib, Gary L. Gallia, Andreas Linninger, Fady T. Charbel, Ankit I. Mehta

**Affiliations:** 1 Department of Neurosurgery, University of Illinois at Chicago, Chicago, IL 60612 United States; 2 Department of Bioengineering, University of Illinois at Chicago, Chicago, IL 60607 United States; 3 Department of Neurosurgery, Johns Hopkins University School of Medicine, Baltimore, Maryland United States

## Abstract

Most applications of nanotechnology in cancer have focused on systemic delivery of cytotoxic drugs. Systemic delivery relies on accumulation of nanoparticles in a target tissue through enhanced permeability of leaky vasculature and retention effect of poor lymphatic drainage to increase the therapeutic index. Systemic delivery is limited, however, by toxicity and difficulty crossing natural obstructions, like the blood spine barrier. Magnetic drug targeting (MDT) is a new technique to reach tumors of the central nervous system. Here, we describe a novel therapeutic approach for high-grade intramedullary spinal cord tumors using magnetic nanoparticles (MNP). Using biocompatible compounds to form a superparamagnetic carrier and magnetism as a physical stimulus, MNP-conjugated with doxorubicin were successfully localized to a xenografted tumor in a rat model. This study demonstrates proof-of-concept that MDT may provide a novel technique for effective, concentrated delivery of chemotherapeutic agents to intramedullary spinal cord tumors without the toxicity of systemic administration.

## Introduction

Nanoparticle technology is emerging as a novel approach for the treatment of cancer^1^. Currently, treatment protocols for most cancers involve surgical biopsy or resection, followed by radiation or chemotherapy. While chemotherapy is effective for some tumors, it is limited by poor specificity and is often unable to cross the blood-spine barrier. Many chemotherapeutic agents further exhibit significant systemic toxicity and a narrow therapeutic index^2^. Nanoparticles have been demonstrated to be a potential solution for these challenges, and are able to enhance chemotherapy efficacy by increasing drug concentration at the target site^3^. To date, five nano-carriers have been approved by the U. S. Food and Drug Administration, four by international agencies, and many others are undergoing clinical investigation and development^1^.

Spinal cord tumors represent a significant challenge in oncology, and account for 2–10% of all central nervous system (CNS) tumors^4^. Intramedullary spinal cord tumors (IMSCTs) account for 8–10% of all spinal cord tumors with astrocytoma being the most common among adolescents and children^4^. While most IMSCTs are benign, 7–30% of astrocytomas are considered malignant and carry a mean survival of 15.5 months^5^.

The unique challenge of IMSCTs is the lack of a clear plane of dissection, making gross total resection hazardous. While some lesions have an identifiable plane of dissection, making gross-total resection (GTR) feasible^6,7^, other lesions, such as astrocytomas and anaplastic ependymomas tend to be infiltrative, making GTR challenging without risking neurologic deficit^8–10^. In cases where GTR is not possible, adjuvant radiotherapy is often recommended, but has significant detrimental side effects, especially in children. Radiation necrosis, myelopathy, developmental deformities, impaired growth, vasculopathy, and parenchymal changes have each been reported following radiation, as well as up to a 25% risk of developing a secondary tumor in 30 years^4,11,12^. Chemotherapy is recommended for certain high-grade IMSCTs, but carries several disadvantages as well. Most chemotherapeutic agents have limited penetration through the blood-spine barrier, are poorly specific, and exhibit significant systemic toxicity, particularly in children^12,13^. Intrathecal drug delivery has been demonstrated to be an effective approach for bypassing the blood spinal cord barrier, but penetration of the spinal cord parenchyma remains a challenge^14–17^. The difficulty in treating IMSCTs makes novel treatment delivery paradigms and convection enhanced delivery attractive alternatives to traditional therapies. To demonstrate proof of concept, this study illustrates a novel treatment for high-grade IMSCTs using doxorubicin-loaded magnetic nanoparticles (DOX-MNPs) guided by a magnetic field.

## Results

### Establishing an Orthotopic Xenograft IMSCT Model

**Figure 1:**
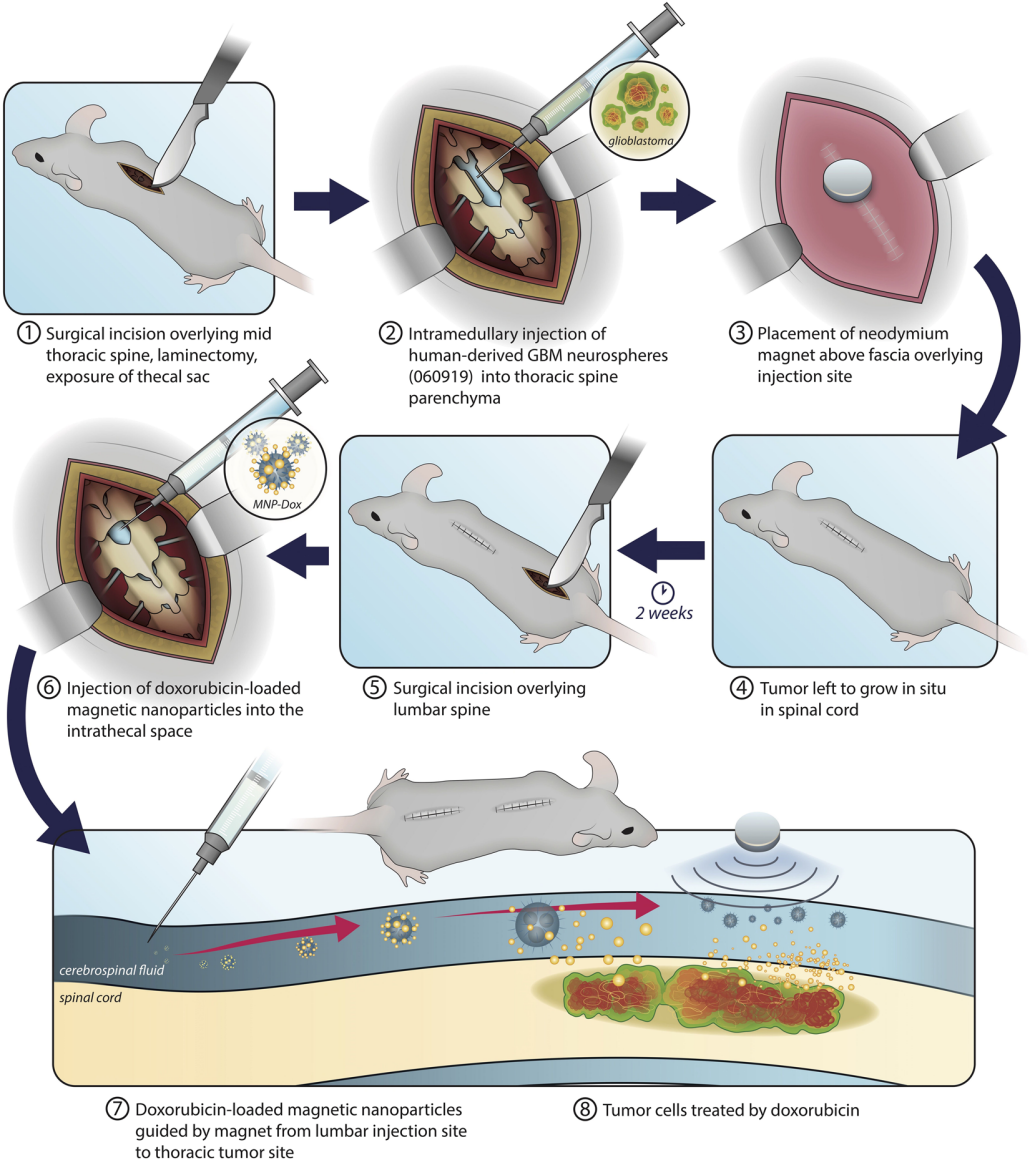
Schematic of experimental design. An initial thoracic incision and laminectomy were performed for inoculation of GBM cells, followed by subdermal placement of a neodymium magnet. Tumor cells were left for two weeks to grow *in situ*, after which the thoracic spine was exposed and MNP-Dox nanoparticles were introduced into the lumbar intrathecal space. The previously implanted magnet then guided the nanoparticles to the tumor site. Image illustrated by Victoria Zakrzewski.

**Figure 2:**
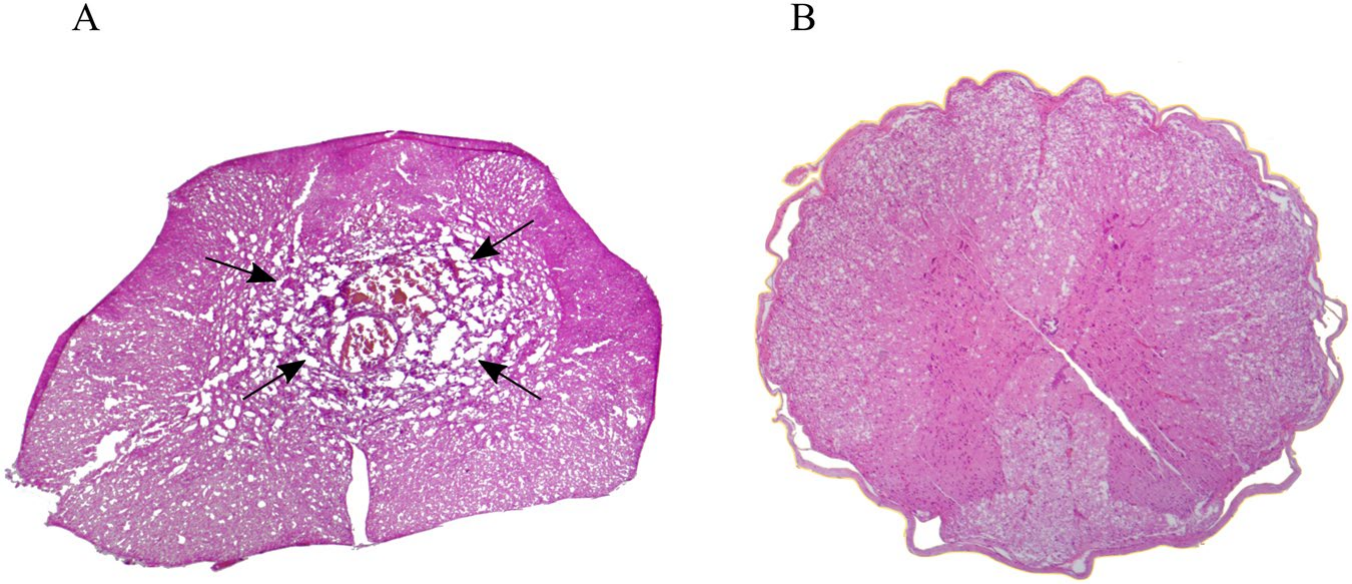
H&E axial spinal cord section three weeks after tumor injection. (**A**) Highly cellular lesion with atypia and central necrosis (arrows) was observed with local invasion of the surrounding parenchyma. (**B**) Comparison with normal spinal cord without tumor injection.

A previously established protocol^18^ was adapted to create a rat intramedullary spinal cord tumor model. Six athymic rats were inoculated with 100,000 human glioblastoma multiforme cells (GBM; Line 060919^19^) into the spinal cord and a neodymium magnet was implanted sub-dermally above the tumor inoculation site. The tumor was allowed to grow for 2 weeks. During this time, 3 rats received an intrathecal injection of DOX-MNPs at the L3/L4 vertebral level. All rats were then sacrificed at week 3 and the spinal cords were removed (Fig. 1). Only 2 rats from the control group and 1 rat from the treatment group showed intramedullary tumor growth. In the other rats, the tumor either failed to grow or began growing in other parts of the spinal cord. The weight of the animals was measured regularly and remained constant throughout the experiment. Fixed frozen tumor sections were prepared and stained with hematoxylin and eosin (H&E) to analyze tumor integrity. Histopathological analysis showed a high-grade infiltrative lesion originating from intramedullary spinal cord parenchyma (Fig. 2).

### Delivery and Localization of Doxorubicin-Loaded Magnetic Nanoparticles

**Figure 3:**
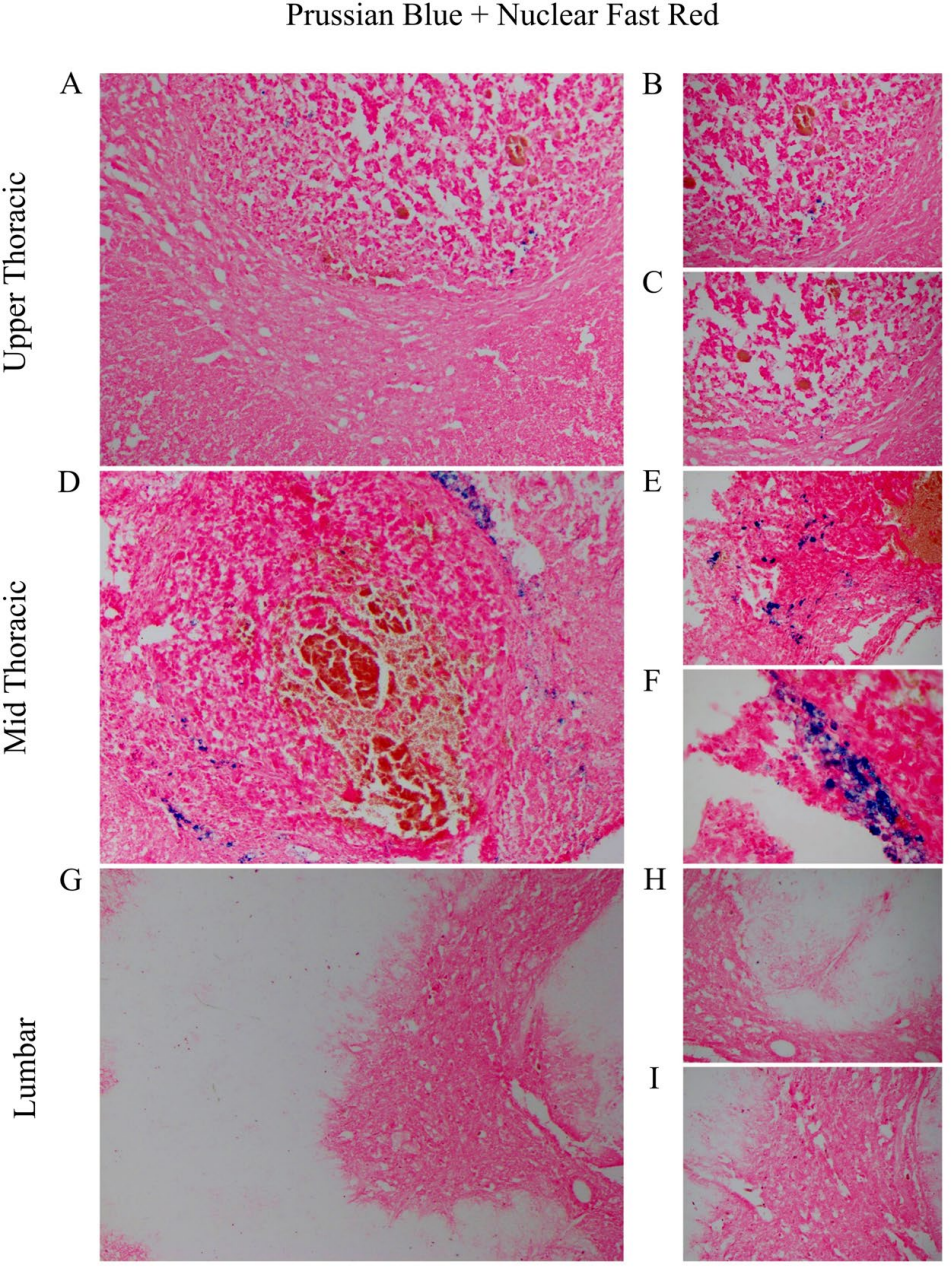
Magnetic Nanoparticle (MNP) Localization at the Tumor Site. (**A**–**D**) MNPs were noted penetrating tumor parenchyma at the upper-thoracic and mid-thoracic level (100x). (**B**–**C**, **E**–**F**) Higher magnification of MNPs penetrating tumor parenchyma (200X). (**G**) MNPs were not detected in the spinal cord at the lumbar control level (100X). (**H**–**I**) Higher magnification revealed no evidence of MNPs at the lumbar level (200X).

Following surgical inoculation of tumor cells, a neodymium magnet was implanted sub-dermally overlying the laminectomy site for the treatment group to create a magnetic field at the site of the tumor and serve as physical stimulus. Continuous fixed frozen sections of the entire spinal cord were prepared and stained using Prussian blue for iron and counterstained with fast red, to visualize the localization of DOX-MNPs within the spinal cord. Histopathologic analysis identified that magnetic nanoparticles were successfully localized at the tumor site, with no magnetic nanoparticles detected at a lumbar control level (Fig. 3).

### Drug uptake and intracellular localization

**Figure 4:**
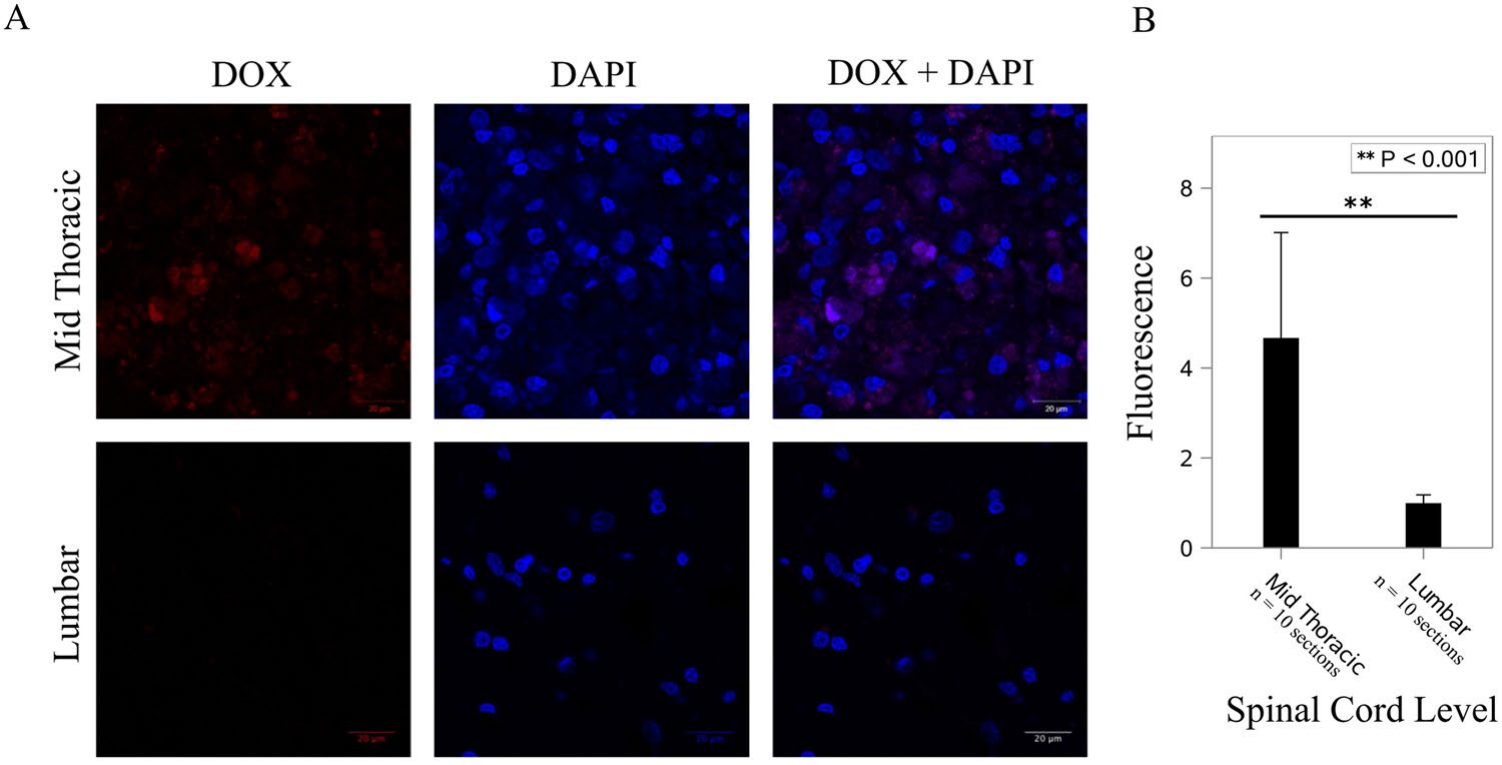
Doxorubicin Localization at the Tumor Site. (**A**) Doxorubicin (ex:480 nm/em:560 nm) was detected in the thoracic level, corresponding to magnetic targeting and co-localized in cell nuclei stained by DAPI. (**B**) Greater doxorubicin fluorescence was noted at the thoracic level versus the lumbar control level (*p* < 0.001).

**Figure 5:**
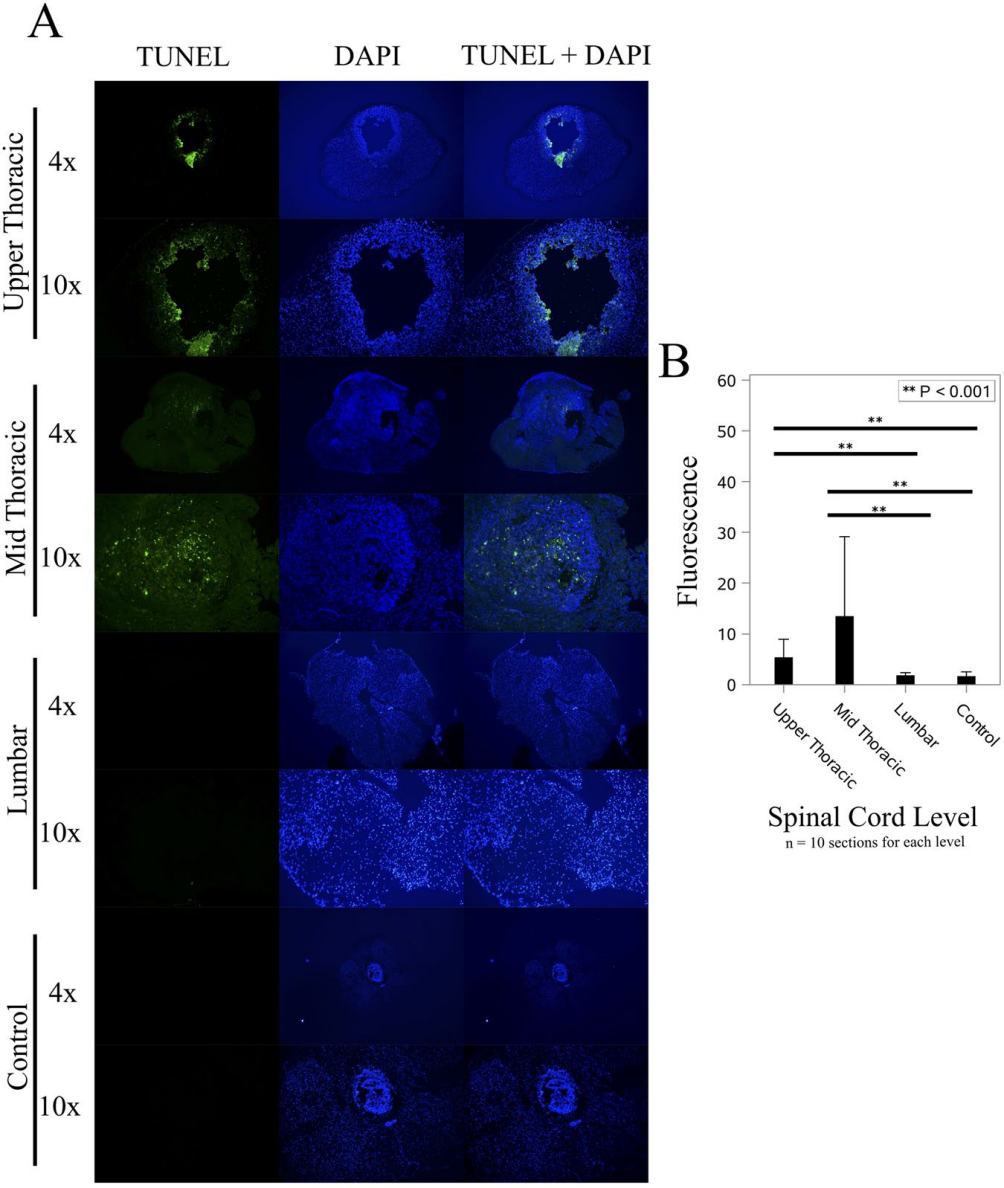
DOX-MNPs Induced Apoptosis in Tumor Cells. (**A**) In treated rats, apoptotic, TUNEL positive cells (green) were localized to the tumor in the upper and mid thoracic spine, and absent in the normal spinal cord of the lumbar spine. No significant apoptotic, TUNEL positive cells were noted in the tumor parenchyma of untreated rats. (**B**) Significantly greater TUNEL florescence was noted in the treated tumor parenchyma (*p* < 0.001).

**Figure 6:**
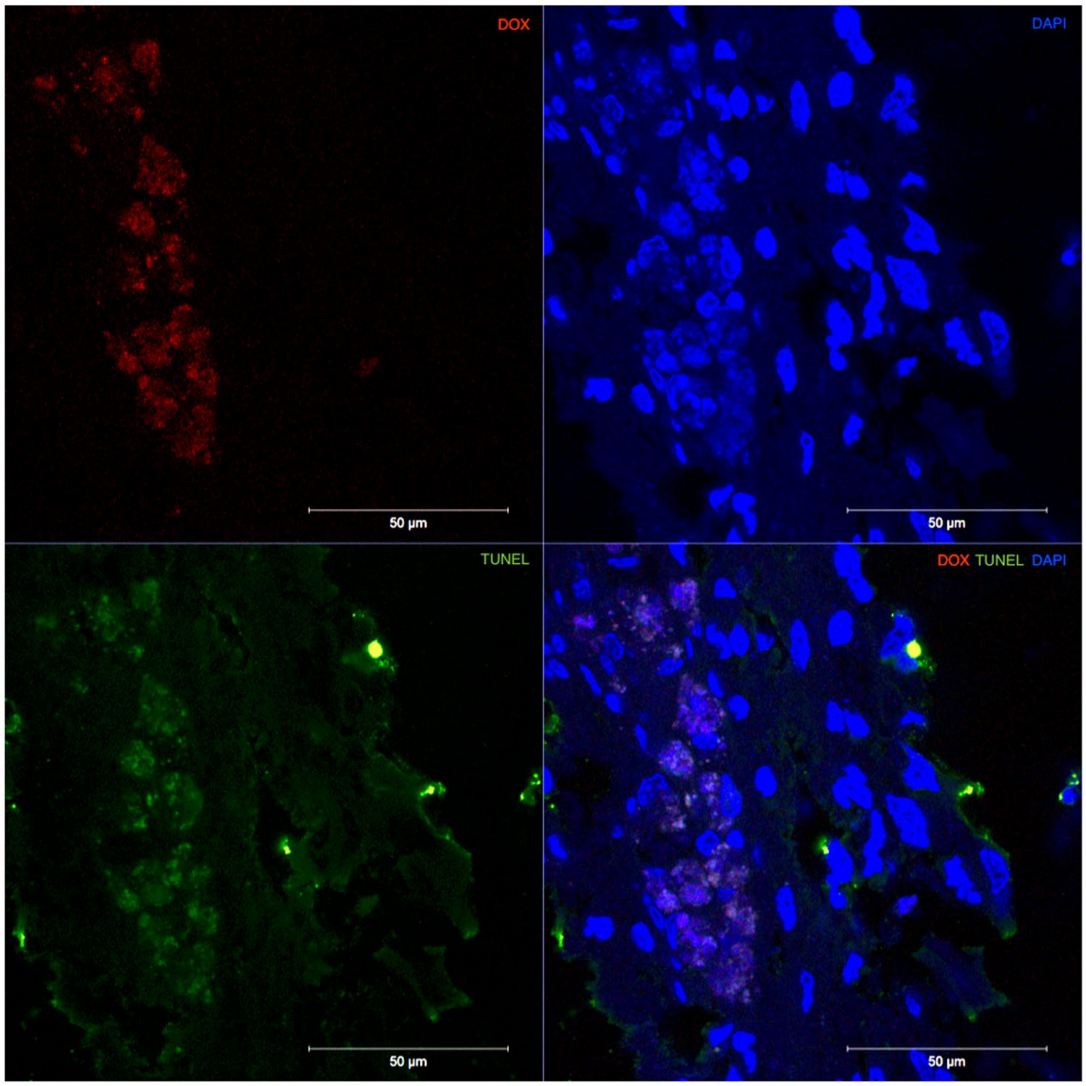
DOX-MNPs & TUNEL Co-Localization. Doxorubicin (red) co-localized with TUNEL positive cells (green) in the tumor parenchyma of the treatment group.

Cellular uptake and intracellular localization of DOX-MNPs was investigated by fluorescence microscopy based on the intrinsic fluorescence of doxorubicin. One week after intrathecal injection of DOX-MNPs, rats were sacrificed and fixed frozen sections were prepared and mounted using Vectashield with DAPI. Using spectral unmixing (ZEN 2012), doxorubicin was found to have the strongest emission peak at 558 nm with an absorption of 488 nm. Confocal microscopy was then used to evaluate the cellular uptake of doxorubicin. Doxorubicin fluorescence was found to be localized to the tumor site at midthoracic level corresponding to the magnetic field and tumor, and was not detected at the control lumbar level (Fig. 4). Fluorescence intensity statistics between the mid-thoracic and lumbar levels revealed significantly greater fluorescence at the thoracic level (*p* < 0.001). Doxorubicin was found to be taken up and localized intracellularly to the cell nucleus, with prominent DAPI co-localization. To evaluate the ability of DOX-MNPs to induce apoptosis in xenografted human tumors, TUNEL staining of spinal cord segments containing tumor was performed. Treated tumors revealed a significant quantity of TUNEL-positive cells (*p* < 0.001) (Fig. 5), which co-localized with DOX-MNPs in both the central tumor mass and pedicles of infiltrating tumor within healthy spine (Fig. 6). Negligible apoptotic activity was noted in other spinal cord segments of the treatment rat, and in untreated tumors of control rats (Fig. 5).

## Discussion

Treatment of high-grade intramedullary spinal cord tumors (IMSCTs) presents a significant challenge to the spinal oncology community^4,20^. IMSCTs carry a poor prognosis with a mean survival of 15.5 months^5^. Given their infiltrative nature and the challenge in obtaining a clear plane of resection, GTR of these lesions is rarely possible without placing spinal motor and sensory pathways at risk^6,7^. Since these high-grade lesions are commonly found in the adolescent population, radiotherapy is usually not recommended due to toxicity to the developing central nervous system^4,11,13^. Chemotherapy therefore is a central part of treatment, but is limited by systemic toxicity, poor drug delivery, and limited tumor parenchyma penetration. Given the limits of chemotherapy, novel therapeutic approaches are needed for IMSCT treatment^12^. In this study, we demonstrate, for the first time in spinal tumors, proof-of-concept that chemotherapeutic drugs can be delivered locally to a tumor site using magnetic nanoparticles guided by a magnetic field.

Magnetic drug targeting has potential as an alternative treatment for IMSCTs by allowing chemotherapeutic agents, administered intrathecally, to bypass the blood-spine barrier and be directed to a target site by magnet. In this study, significantly more nanoparticles and doxorubicin were localized at the mid-thoracic level where the tumor and magnetic field was applied compared to other spinal cord levels. The efficacy of DOX-MNPs to induce cellular apoptosis in tumor cells was previously shown *in vitro*^21–23^. Here, apoptosis was shown to be co-localized with doxorubicin, confirming the therapeutic effectiveness of MNP-DOX *in vivo*. Negligible apoptosis was detected at the lumbar level, indicating low CNS toxicity.

A number of drug delivery carriers have been explored for targeted drug delivery. Although some show potential for medical applications, the use of magnetic nanoparticles has many advantages when considered for targeted drug delivery applications^24^. Magnetic nanoparticles can be manipulated under the influence of an external magnetic field, as a result of superparamagnetisim^25,26^, offering the advantage of reducing particle aggregation in locations other than where the magnetic field is present^25^.

MNPs in this study consisted of chemicals composed of iron oxide, which is biodegradable, biocompatible, and demonstrates a chemical stability of higher quality compared to other metallic nanoparticles^27^. DOX-MNPs are uniquely engineered and optimized for cancer cell targeting^28–33^. By synthesizing the DOX-MNPs to an average size of 100 nm, the vehicle system is small enough to be suitable for systemic and intrathecal delivery, but large enough to be easily taken up by cells^21,34–37^. Having a larger surface area-to-volume ratio provides the advantage of customizing the surface with a specific drug, which can lead to faster drug release compared to an encapsulated drug^38,39^. The surfaces of the MNPs used in this study allow for a strong electrostatic bond to take place between the negatively charged carboxylate groups of gellan gum and the positively charged amine group of doxorubicin. Once DOX-MNPs reach the acidic tumor microenvironment, the electrostatic interaction is broken, releasing doxorubicin. Different chemotherapeutic agents can be attached to MNPs by using different polymer coats or altering the functional groups of these agents, making MNPs a versatile mechanism applicable in the treatment of various malignancies.

### Future Directions

By increasing the concentration of doxorubicin at the tumor site, it is possible to improve the chemotherapeutic index and improve the efficacy and safety of treating IMSCTs. Subsequent follow up studies should include larger sample sizes, assess the effect of therapy on decreasing the rate of tumor growth, preventing the onset of neurologic deficits, and improving survival. Future studies may further compare intrathecal treatment with DOX-MNPs with standard, systemic treatment to identify differences in outcomes and undesirable side effects. If results demonstrate the utility of MNPs in reducing toxicity, decreasing tumor growth, and improving survival, a limited clinical trial may be considered as a next step.

### Limitations

Although this study began with several athymic rats, treatment results are based on a single animal. Several challenges persist in the inoculation of the animals with tumors and injection of MNP-DOX into the intrathecal space. Because of the small size of the rat spinal cord, precise needle placement and infusion is necessary to successfully inoculate tumors and inject MNP-DOX. In one rat, injection did not fully penetrate into the thecal space, leading to no tumor growth within the spinal cord parenchyma. In another rat, MNP-DOX was not correctly injected into the intrathecal space. In another rat, unexpected death was encountered shortly after surgery. While only one animal successfully underwent tumor inoculation, treatment and localization of MNPs was successful in this subject, providing proof of concept. There were no instances of the inability to localize MNPs when rats survived the length of the study and both tumor inoculation and MNP injection were successful.

## Conclusions

Targeted delivery of nanoparticles holds promise in treating invasive cancers. For intramedullary spinal cord tumors, targeted delivery can reduce systemic and CNS toxicity as well as increase therapeutic index by localizing chemotherapy at a specific spinal cord level. The results of this study provide proof of concept and demonstrate focal, chemotherapy-induced apoptosis of tumor cells by localization of DOX-MNPs *in vivo* using a magnetic field.

## Materials and Methods

### Generation of an Orthotopic Rat Xenograft Tumor Model

Development of the tumor model is summarized in Fig. 1. The protocol established by W Hsu *et al*.^18^ for generating an animal model for intramedullary spinal cord glioma was adapted. Immunodeficient athymic nude rats Crl:NIH-*Foxn1*^*rnu*^ (Charles River Laboratories) weighing 200–300 grams were obtained. Rats were first anesthetized with 1–3% isoflurane in a gas chamber followed by continuous delivery of isoflurane via nose-cone; anesthesia was monitored by continuous observation of respirations and toe pinch every 15 minutes. The dorsal midthoracic region was shaved and cleaned with 3 alternating washes of alcohol and Povidone/Iodine solution. The spinous processes at midthoracic level were identified by palpation before a 2 cm longitudinal midline incision was made through the skin. After hemostasis was achieved using bipolar electrocautery, the underling fascia and paravertebral muscles were retracted laterally with a cotton tip applicator and self-retraining retractor. A rongeur was then used to remove the lamina and spinous process of a single level, followed by the ligamentum flavum. Once the dura was identified, hemostasis was again achieved and a small incision made using a 27G needle. The needle was advanced and the location of the dorsal spinal cord confirmed by lower-extremity motor reflex. 100,000 human glioblastoma multiforme (GBM; Line 060919^19^) neurospheres in 3 microliters of Dulbecco’s medium modified eagle medium (DMEM) were inoculated into the spinal cord over 1 minute to minimize cell extravasation. Following the injection, the needle was left in place for 5 minutes and then slowly retracted. A 1 cm, 0.01 Tesla strength neodymium-iron-boron (NdFeB) magnet was then implanted sub-dermally over the laminectomy site and the incision closed using inverted vicryl sutures for the dermis and running nylon sutures for the epidermis. One dose of analgesia (Buprenorphine SR lab 1.0 mg/kg) was administered intraperitoneally postoperatively. Sutures were removed 10 days after surgery. The implanted tumors were then allowed to grow for a period of two weeks. All experiments involving animals were approved by the Animal Care and Use Committee of the University of Illinois at Chicago and conducted in accordance with the guidelines and regulations of the ACUC.

### Magnetic Nanoparticle Synthesis and Delivery

Doxorubicin-loaded magnetic nanoparticles used in this study were synthesized according to a previously published technique by Venugopal *et al*.^26^. The formulation of these particular MNP’s begins with creation of iron oxide magnetite (Fe_3_O_4_) cores by coprecipitation. Once iron cores were created, an outer layer of gold was added onto the surface of these cores, serving as an inert protective layer and a platform for the polymer gellan gum. Electrostatic interactions between gellan gum and doxorubicin allowed for loading of the chemotherapeutic drug resulting in DOX-MNPs. The DOX-MNPs were resuspended in water to concentration of 40 mg mL^−1^. Each step was carried out under aseptic conditions.

Following a two-week incubation period, the doxorubicin-loaded magnetic nanoparticles were administered. Rats were anesthetized, positioned, and prepped in the same manner described previously. A 2 cm longitudinal midline incision was made through the skin at the L3 and L4 vertebral level. The underling fascia and paravertebral muscles were then retracted laterally using with a cotton tip applicator and self-retraining retractor, and the lamina was exposed. A 27G needle attached to a Hamilton syringe was introduced through the intervertebral space between vertebral level L3 and L4. An injection-induced tail-flick and decrease in pressure confirmed insertion of the needle into the intrathecal space. Fifteen microliters of DOX-MNPs were then administered over 1 minute in the experimental group and 15 microliters of 1X solution of phosphate-buffered saline (PBS) in the control group. Once injection was completed, the needle was left in place for 5 minutes and then slowly retracted. Animals were then euthanized one week after DOX-MNP administration for analysis.

### Histopathological Analysis

All histologic procedures were performed in the dark to ensure minimal light exposure and avoid photo bleaching. After euthanasia, the spinal cord was removed and fixed in 4% formalin in PBS overnight. Tissue was then transferred to 30% sucrose in 1X PBS and left overnight. After sucrose cryoprotection, 2 mm axial sections were cut and acclimated in Optimal cutting temperature compound (OCT) for a few minutes. Samples were transferred to cryomolds, covered with OCT, rapidly frozen using a dry ice methanol slurry bath, and stored in at −80 °C. Frozen sections (12 µm; Leica CM 1860 UV) were obtained and mounted on slides. H&E and Prussian blue stains counterstained with fast red (IHCworld protocol) were performed and analyzed using a Zeiss optical microscope. Samples were mounted with Vectashield with DAPI to observe localization of MNP-DOX. Tumor cell apoptosis was evaluated by the Click-iT TUNEL Alexa Fluor 597 Imaging Assay (Invitrogen) according to manufacturer instructions. Spectral unmixing (ZEN 2012, Zeiss LSM 710 BIG) was used to identify the peak emission of doxorubicin in the tumor microenvironment. Ten random fields were selected for statistical analysis using Fiji, with high intensity spots as the fluorescence signals of MNP-DOX or TUNEL, and low intensity fluorescence was used as the background noise^40,41^. Photos were taken using a confocal microscope (Zeiss LSM 710) and a fluorescent microscope (EVOS FL Auto 2 Cell Imaging System).

### Statistical Analysis

An independent samples *t-*test with unequal variances was conducted to compare doxorubicin fluorescence intensities between the midthoracic and lumbar levels. Kruskal-Wallis test followed by a Dunn’s multiple comparison test was performed on TUNEL fluorescence intensities among midthoracic, lumbar, and the control group. The significance level was set to alpha 0.05.
